# Imaging Mass Spectrometry Detection of Gangliosides Species in the Mouse Brain following Transient Focal Cerebral Ischemia and Long-Term Recovery

**DOI:** 10.1371/journal.pone.0020808

**Published:** 2011-06-08

**Authors:** Shawn N. Whitehead, Kenneth H. N. Chan, Sandhya Gangaraju, Jacqueline Slinn, Jianjun Li, Sheng T. Hou

**Affiliations:** 1 Experimental NeuroTherapeutics Laboratory, Institute for Biological Sciences, National Research Council of Canada, Ottawa, Ontario, Canada; 2 Mass Spectrometry Glycoanalysis Laboratory, Institute for Biological Sciences, National Research Council of Canada, Ottawa, Ontario, Canada; 3 Department of Biochemistry, Microbiology and Immunology, University of Ottawa, Ottawa, Ontario, Canada; National Institute on Aging Intramural Research Program, United States of America

## Abstract

Gangliosides, a member of the glycosphingolipid family, are heterogeneously expressed in biological membranes and are particularly enriched within the central nervous system. Gangliosides consist of mono- or poly-sialylated oligosaccharide chains of variable lengths attached to a ceramide unit and are found to be intimately involved in brain disease development. The purpose of this study is to examine the spatial profile of ganglioside species using matrix-assisted laser desorption/ionization (MALDI) imaging (IMS) following middle cerebral artery occlusion (MCAO) reperfusion injury in the mouse. IMS is a powerful method to not only discriminate gangliosides by their oligosaccharide components, but also by their carbon length within their sphingosine base. Mice were subjected to a 30 min unilateral MCAO followed by long-term survival (up to 28 days of reperfusion). Brain sections were sprayed with the matrix 5-Chloro-2-mercaptobenzothiazole, scanned and analyzed for a series of ganglioside molecules using an Applied Biosystems 4800 MALDI TOF/TOF. Traditional histological and immunofluorescence techniques were performed to assess brain tissue damage and verification of the expression of gangliosides of interest. Results revealed a unique anatomical profile of GM1, GD1 and GT1b (d18∶1, d20∶1 as well as other members of the glycosphingolipid family). There was marked variability in the ratio of expression between ipsilateral and contralateral cortices for the various detected ganglioside species following MCAO-reperfusion injury. Most interestingly, MCAO resulted in the transient induction of both GM2 and GM3 signals within the ipsilateral hemisphere; at the border of the infarcted tissue. Taken together, the data suggest that brain region specific expression of gangliosides, particularly with respect to hydrocarbon length, may play a role in neuronal responses to injury.

## Introduction

Gangliosides are a member of the glycosphingolipid family and consist of mono- to poly-sialylated oligosaccharide chains attached to a ceramide base. Gangliosides are amphiphilic molecules found within the outer layer of the plasma membrane of all vertebrate cells [1] but are particularly enriched within the plasma membrane of the central nervous system and account for 1% of the total lipid weight within the brain [2], [3]. The main gangliosides within the adult human brain are GM1, GD1a, GD1b and GT1b [4].

Gangliosides, specifically GM1, have been identified as a major lipid constituent of membrane rafts in many cell types [5], [6]. Gangliosides have a wide range of CNS functions including membrane function [7], axon stability and regeneration [8], differentiation [9] and neurodegeneration [10]–[12]. The ceramide moieties of gangliosides are also heterogeneous with respect to carbon chain length within their sphingosine base. It has been suggested that d18∶1 and d20∶1 gangliosides are differentially regulated and may possess unique roles with respect to neuronal function and cell death [13], [14].

Altered ganglioside synthesis and its role in neurodegeneration have garnered recent attention. Studies have shown that mice deficient in glycosyltransferases, enzymes that are responsible for the synthesis of gangliosides, exhibited degeneration within both the PNS and CNS. Accumulations of GM2 and GM3, precursors to GM1, can occur in the absence of proper ganglioside synthesis. High levels of GM2 and GM3 have been implicated in lysosomal disorders and lead to neurodegeneration [11], [15], [16].

Imaging mass spectrometry (IMS) using matrix-assisted laser desorption/ionization (MALDI-TOF) is a powerful tool for visualizing the spatial distribution of various molecules *in situ* [17]. IMS has been used to examine the spatial profiling of lyso-phosphatidylcholine following stroke in the rat [18]. IMS has also been used successfully to examine the spatial profile of gangliosides and other glycosphingolipids in the mouse brain during development [13], [14]. The objective of this study was to examine the temporal and spatial profile of ganglioside species using IMS following middle cerebral artery occlusion (MCAO) and long-term reperfusion injury in the mouse. This is a potent method to not only discriminate gangliosides by their oligosaccharide components, but also by their hydrocarbon lengths. Here we describe a unique anatomical profile of GM1, GD1 and GT1b (d18∶1, d20∶1) as well as other members of the glycosphingolipid family. There was marked variability in the ratio of expression between ipsilateral and contralateral cortices for the various detected ganglioside species following MCAO-reperfusion injury. Interestingly, MCAO resulted in the transient induction of both GM2 and GM3 signals within the ipsilateral hemisphere, at the border of the infarcted tissue. Immunofluorescently labeled brain tissue sections with antibodies against GM1, GD1a and GM3 revealed similar expression profiles to those observed using IMS. Taken together, the data suggest that brain region specific regulation of gangliosides, particularly with respect to sphingosine base length, may play a role in neuronal responses to injury.

## Results

### Imaging MALDI-TOF detection of gangliosides within the mouse brain

**Figure 1:**
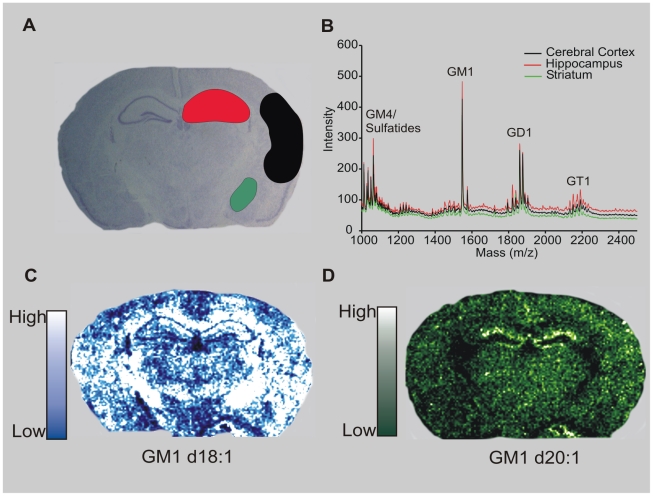
Imaging of gangliosides within a mouse brain using MALDI-TOF. (A): Sham-operated mice were sacrificed and their brains were removed, flash frozen and sectioned on a freezing cryostat. ROIs were determined from adjacent 10 µm thick mouse brain sections stained with Cresyl violet. (B): Total ion chromatograph depicting the mass spectra from the highlighted ROIs. Peaks were identified corresponding to GM4/sulfatides, GM1, GD1 (a and b) and GT1b. (C): IMS of a coronal mouse brain section corresponding to GM1 d18∶1 (m/z 1546); and GM1 d20∶1 (m/z 1574) (D). Note the unique neuroanatomical profiles, specifically within the hippocampus for each species. Colored bars in C and D represent relative levels of MALDI-TOF signal.

Adjacently sectioned coronal mouse brain sections were stained with Cresyl Violet to determine the neuroanatomical regions to be selected for regions of interest (ROIs). ROIs included the cerebral cortex, striatum and hippocampus (Figure 1A). Mass spectrum from each ROI was extracted and plotted (Figure 1B). In negative ion mode sulfatides (1000–1200), GM1 (1545, 1573), GD1 (comprising both GD1a and GD1b species which cannot be distinguished by MALDI-TOF; 1800–1900), and GT1b (2150–2250) were detected. For GM1, both the d18∶1 and d20∶1 moieties were detected (Figure 1 C,D). For GD1 and GT1b d18∶1 and d20∶1 species were also detected along with their sodium (GD1 d18∶1 Na^+^ - 1860; GD1 d20∶1 Na^+^ - 1888, GT1b d18∶1 Na^+^ - 2151, GT1b d20∶1 Na^+^ - 2179) and potassium (GD1 d18∶1 K^+^ - 1876; GD1 d20∶1 K^+^ - 1904, GT1b d18∶1 K^+^ - 2167, GT1b d20∶1 K^+^ - 2195) adducts. No sodium or potassium adducts were detected with GM1 species. The sodium and potassium adducts for GD1 and GT1b but not GM1 were in confirmation with previous studies [13], [14].

### Detection and quantification of GM1 following MCAO and long-term reperfusion in the mouse

**Figure 2:**
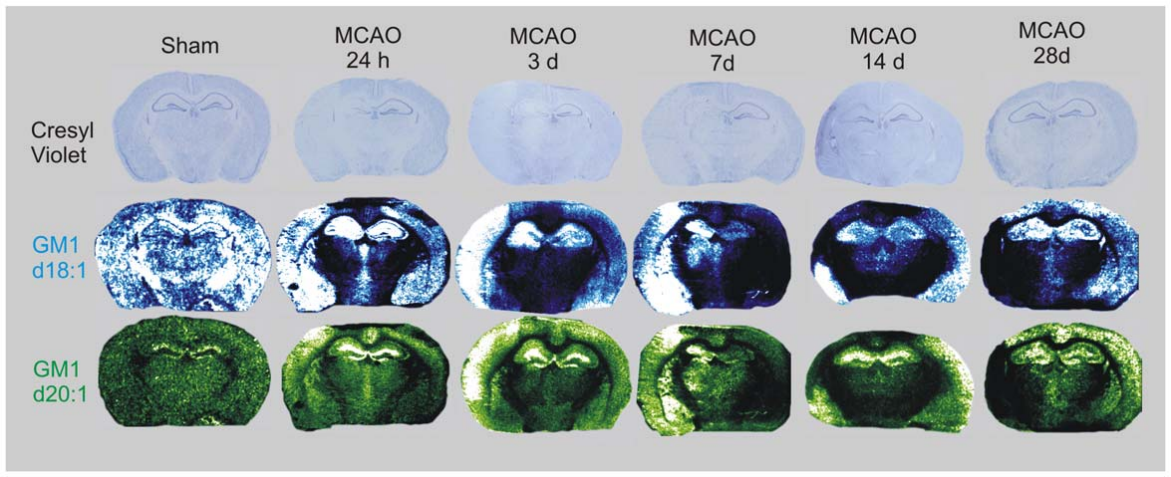
GM1 imaging within a mouse brain following a unilateral MCAO and long-term survival. Mice received a 30 min unilateral MCAO followed by reperfusion time points of 24 h, 3 d, 7 d, 14 d and 28 d. Adjacent sections were either stained with Cresyl violet; or were scanned using mass spectrometry imaging. Mass spectrometry images for each time point were from the same coronal tissue section. Both GM1 d18∶1 (m/z 1546) and d20∶1 (m/z 1574) were expressed at higher levels within the MCAO-induced infarcted hemisphere, specifically within the cerebral cortex, hippocampus and striatum.

Both GM1 d18∶1 and d20∶1 species were detected in multiple brain regions (Figure 2) including the cerebral cortex, hippocampus and striatum. Sham-operated mice demonstrated high levels of GM1 d18∶1 throughout the brain; whereas d20∶1, although detectable in small amounts throughout the brain, was mainly detected within the dentate gyrus of the hippocampus. The expression of GM1 d18∶1 and GM1 d20∶1 differed following MCAO and long-term reperfusion. At 24 h and 3 d post-MCAO, GM1 d18∶1 signal was up-regulated within the ipsilateral cortex, striatum and hippocampus and the level of GM1 d18∶1 peaked by 7 d post-MCAO. By 14 d, the signal had dropped within the ipsilateral hemisphere within brain areas where tissue viability had been lost. GM1 d20∶1 was up-regulated at 24 h and peaked by 3 d post-MCAO within the ipsilateral cortex and in the hippocampi of both sides of the hemisphere. By 7 d post-MCAO, GM1 d20∶1 was restricted to regions surrounding the infarct core, as determined by Cresyl violet staining. It is important to note that in each time point, the imaging depicts relative signal between ipsilateral and contralateral hemispheres. For example, even though little signal is visually shown in the contralateral hemisphere of 3 d and 7 d post-MCAO, a concrete mass spectrum can still be derived.

**Figure 3:**
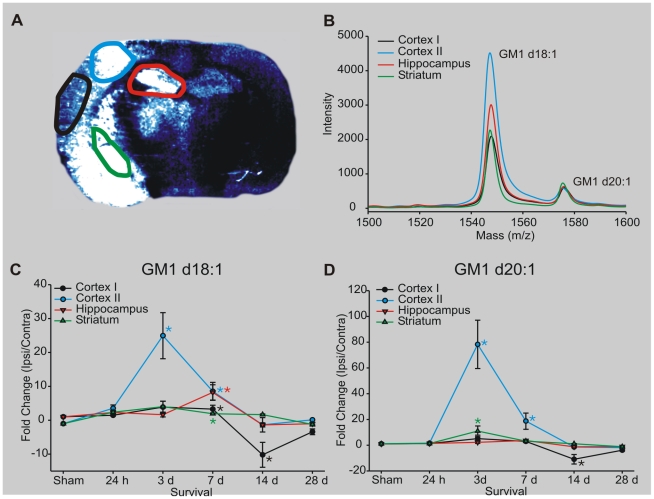
Quantifying GM1 species within the mouse brain following MCAO and long-term survival. (A): IMS of GM1 d18∶1 from a mouse receiving a MCAO followed by 7 d reperfusion. ROIs were highlighted as shown. (B): Mass spectra were extracted from ipsilateral and contralateral ROIs. (C,D): The area underneath the total ion chromatograph curve was calculated and plotted. Data represents the mean ratio of signal intensity between ipsilateral and contralateral ROIs; * indicates p<0.05, n = 3.

To quantify the relative changes in GM1 d18∶1 and d20∶1 between the ipsilateral and contralateral sides of the brain, ROIs were chosen based on Cresyl violet staining of adjacent brain sections and confirmed using a mouse brain atlas [19]. These regions included two measurements in the infarcted cerebral cortex, a region containing the ventral secondary auditory cortex, somatosensory S1 and S2 cortices and granular insular cortex (cortex I); the area adjacent comprising primary motor, somatosensory trunk region and dysgranular region (Cortex II), as well as the hippocampus and striatum (Figure 3a). ROI measurements were also made in corresponding neuroanatomical regions of the contralateral hemisphere. The mass spectra were extracted from each ROI and plotted as shown in Figure 3B. Two distinct peaks corresponding to GM1 d18∶1 (m/z 1545) and GM1 d20∶1 (m/z 1573) were easily detected in each ROI. The MALDI signal from each ROI was quantified by integrating the area under each peak and calculating the fold change of intensity of ipsilateral ROI signal to contralateral ROI signal from the corresponding neuroanatomical region. The fold change of ipsilateral to contralateral ROIs following MCAO was plotted for each reperfusion time point for both GM1 d18∶1 (Figure 3C) and d20∶1 (Figure 3D). GM1 increases within the ipsilateral cortex I at 24 h following MCAO and then falls below contralateral levels by 14 d post-MCAO (Figure 3C). GM1 d18∶1 signal within the ipsilateral Cortex II peaked by 3 d whereas it peaked by 7 d within the ipsilateral hippocampus. GM1 d18∶1 also increased within the ipsilateral striatum at 24 h, 3 d and 7 d post-MCAO. GM1 d20∶1 level increased most dramatically within the ipsilateral Cortex II region and modestly increased within the ipsilateral hippocampus and striatum by 3 d post-MCAO (Figure 3D).

### Imaging GD1 and GT1b following MCAO reperfusion injury

**Figure 4:**
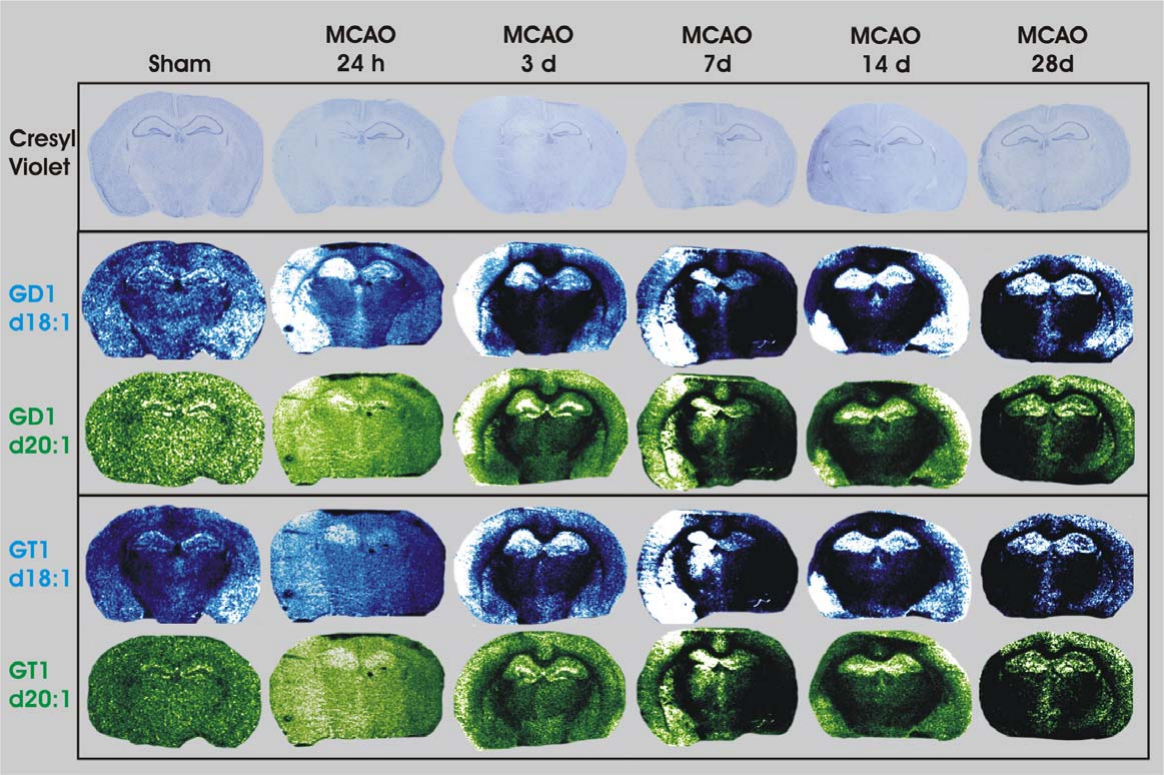
IMS of GD1 (K^+^) d18∶1 (m/z 1876) and d20∶1 (m/z 1904), GT1b (K^+^) d18∶1 (m/z 2167) and d20∶1 (m/z 2195) following MCAO-reperfusion. Both GD1 and GT1 were expressed at higher levels within the MCAO-induced infarcted hemisphere, specifically within the cerebral cortex, hippocampus and striatum.

In order of magnitude, the detected level of relative expression within the non-injured mouse brain was GM1 followed by GD1 and GT1b. All three species demonstrated a similar neuroanatomical expression profile during MCAO-reperfusion. Following MCAO, both GD1 d18∶1 and d20∶1 increased within the ipsilateral cortex (cortex I and II), hippocampus and striatum at 24 h and remained elevated within the cortex II region, hippocampus and striatum through 7 d (Figure 4). By 14 d the level of GD1 was equal within the hippocampus and decreased within the non-viable cortical tissue.

The level of GT1b was detectable within the mouse brain albeit at a low level compared to that of GM1 and GD1 (Figure 4). Following MCAO, both GT1 d18∶1 and d20∶1 increased within the ipsilateral cortex, hippocampus and striatum at 24 h and remained elevated within the Cortex II region, hippocampus and striatum through 7 d. By 14 d the levels of GT1 were equal within the hippocampus while GT1 decreased within the non-viable cortical tissue.

### Detection of GM2 and GM3 within the ipsilateral hemisphere following MCAO

**Figure 5:**
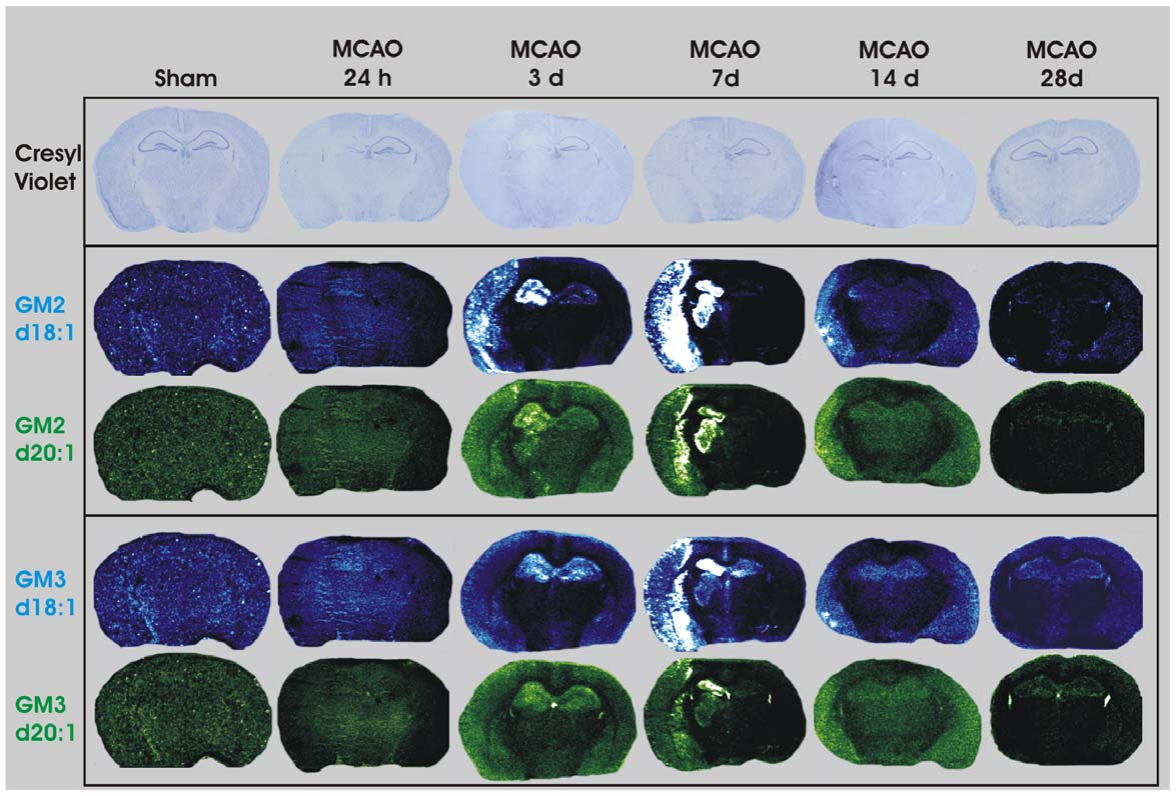
IMS of GM2 d18∶1 (m/z 1384), GM2 d20∶1 (m/z 1412), GM3 d18∶1 (m/z 1181) and GM3 d20∶1 (m/z 1209) following MCAO-reperfusion. Neither GM2 nor GM3 were detected in sham-operated mice or mice receiving MCAO followed by 24 h reperfusion. GM2 and GM3 (d18∶1 and d20∶1) were detected within the cortex, at the periphery of the injured tissue, as well as within the hippocampus, striatum and thalamic structures at 3 and 7 d. Neither GM2, nor GM3 (d18∶1 or d20∶1), were detected in mice by 28 d after MCAO.

Neither GM2 nor GM3 were detected within the brains of uninjured sham-operated mice (Figure 5). Following 3 d post-MCAO, GM2 was up-regulated within the ipsilateral cortex and hippocampus (Figure 5). GM2 remained elevated within these brain regions and spread to the ipsilateral striatum by 7 d post MCAO. There was very little GM2 signal within the mouse brain by 14 d and was completely absent by 28 d post-MCAO. GM3 signal demonstrated a temporal and neuroanatomical profile similar to that of GM2, with the exception that GM3 d18∶1 was detected in both the contralateral and ipsilateral hippocampi at 3 and 7 d post-MCAO whereas GM3 d20∶1 was detected in both the contralateral and ipsilateral hippocampi at 3 d post-MCAO only (Figure 5). The greatest intensity of GM2 and GM3 signal appeared to be at the border of the infarcted tissue based on Cresyl violet staining.

**Figure 6:**
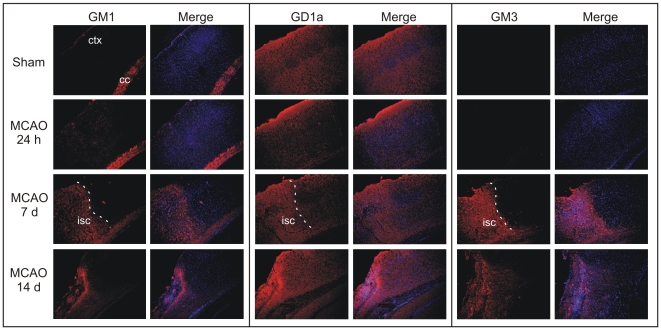
Detection of GM1, GD1a and GM3 by immunofluorescence staining within the mouse brain following MCAO. Mice were perfusion fixed with 4% formaldehyde. Coronal brain sections at 10 µm thickness were cut and immunostained with either mouse anti-GM1, GD1a or GM3 (red color). Sections were counterstained with 2 µg/ml Hoechst 33258 (blue color) and imaged with an Olympus Fluoview FV1000 confocal laser scanning microscope. Photomicrographs depict the border area between ischemic infarct core and the peri-infarct area clearly separated by the white colored line. At 7 d reperfusion, the border of the stroke-induced infarct is visible (lower left half). Expression levels of GM1, GD1a and GM3 increased within the infarcted ipsilateral cortex. GM1 and GM3 increases were restricted to the stroke-induced infarct core (abbreviations: ctx, cerebral cortex; cc – corpus callosum; isc, ischemic area).

To confirm some of the ganglioside expression profiles detected with IMS, GM1, GD1a and GM3 expression were detected by indirect immunofluorescence staining and visualized under a confocal microscope (Figure 6). In the uninjured mouse brain, GM1 was mostly restricted to white matter fiber tracts, whereas GD1a appeared within the grey matter, both of which were confirmed in previous reports [20], [21]. GM3 was not detected by immunofluorescence in any brain region of the uninjured brain, confirming MALDI-TOF data. Both GM1 and GD1a expression increased within the ipsilateral MCAO-damaged cortex at 7 d post-MCAO, and both of which matched the temporal profiles detected by IMS. GM3 expression was dramatically increased within the ipsilateral MCAO-induced cortical tissue; as well as 7 d post-MCAO and appeared to be restricted mostly to gray matter regions. Although the antibodies cannot detect the difference between d18∶1 and d20∶1 moiety, it appears that the temporal expression profile of gangliosides detected by MALDI-TOF and immunofluorescence are fairly similar.

### Summary of Ganglioside expression following MCAO reperfusion injury

**Figure 7:**
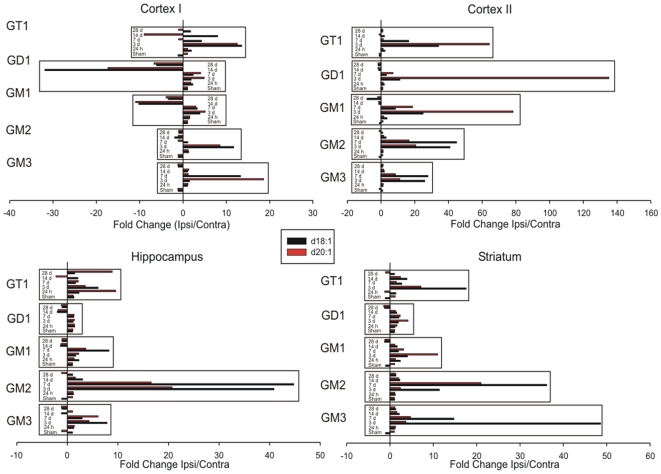
Summary of ganglioside expression with respect to ROIs. The mean intensities of ganglioside derived from the mass spectra were expressed as fold increases/decreases between ipsilateral and contralateral ROIs. Each paired bar represents the d18∶1 (black-colored bar) and d20∶1 (red-colored bar) moieties for each ganglioside species detected. Bars within the box represent the time course (sham, 24 h, 3 d, 7 d, 14 d and 28 d) following MCAO for each ganglioside (GM3, GM3, GM1, GD1 and GT1b).

The fold increases and decreases from ipsilateral to contralateral ROIs were determined for GM1, GD1, GT1b, GM2 and GM3 for both d18∶1 and d20∶1 species following MCAO and various reperfusion time points - sham, 24 h, 3 d, 7 d, 14 d and 28 d. (Figure 7). All four anatomical ROIs (cortex I, cortex II, hippocampus and striatum) demonstrated unique ganglioside profiles with respect to oligosaccharide structure as well as sphingosine (d18∶1 vs. d20∶1) chain length. Generally, the degree of change in d18∶1 and d20∶1 species was relatively similar with a few exceptions. The d20∶1 moiety of GM1, GD1 and GT1b increased at a greater rate within the Cortex II region. Conversely the d18∶1 moiety of GM2 and GM3 increased at a greater rate within the hippocampus and striatum. Also, in most cases, the greatest level of ganglioside change occurred at the 3 and 7 d time points, which were time points associated with secondary neuronal death and neuroinflammation in response to the MCAO.

## Discussion

Gangliosides have been shown to possess a wide variety of functions within the CNS [7]–[11]; however, their expression in the brain following cerebral ischemia and reperfusion has not been well studied. Here, we demonstrate, for the first time, the spatial and temporal profile of gangliosides following MCAO and long-term survival in a mouse model of stroke. The advantage of looking over extended reperfusion time periods is that one can identify several clinically relevant pathological events that follow a stroke. Some of these events include the formation of the necrotic core, delayed neuronal death, scar formation and tissue remodeling.

The transient induction of GM2 and GM3 from 3 to 7 d following MCAO was an intriguing finding. First, neither GM2 nor GM3 could be detected within the uninjured sham brain. The lack of detection does not suggest that no GM2 and GM3 exist within the mouse brain, but does suggest that they were clearly below our level of detection which is consistent with previous studies [4], [14]. The expression of GM2 and GM3 appeared to demonstrate a crescent-like distribution at the border of the infarcted tissue region and extended into regions within the hippocampus and thalamus. Previous reports have demonstrated that accumulations of GM2 and GM3, precursors to GM1, can occur in the absence of proper ganglioside synthesis and that sustained levels of GM2 and GM3 have been implicated in lysosomal disorders and lead to neurodegeneration [11], [15], [16]. Our data may be congruent with this possibility given that GM2 and GM3 expression were seen at the border of the infarcted tissue.

Data obtained using MALDI-MS imaging of gangliosides does have its limitations. Data analysis should be interpreted with caution as studies have demonstrated that sialic acid is susceptible to loss during the MALDI-MS process [14], [22]–[24]. Although we were able to detect GD1 and GT1b species, a significant portion of these species were likely degraded and thus detected as GM1 during the scan. This has been previously addressed in a study examining MS on authentic samples of GM1, GD1 and GT1 gangliosides. Data from this study demonstrated ion signals at m/z 1544 and 1572 that corresponded to ions originating from GM1 and GD1, but not GT1. Ions at m/z 1874 and 1902 (GD1) contained barely traceable levels of GT1-derived signals [14].

One of the distinct advantages of using IMS technology to examine gangliosides is the ability to differentiate species based on their carbon chain length within the sphingosine base. Antibodies specific to the oligosaccharide moiety of gangliosides may be used to visualize individual gangliosides with different oligosaccharide units. However, antibodies cannot detect whether or not these gangliosides have 18 or 20 carbons on their sphingosine base. In any case, there was concurrence for the data obtained by IMS and immunofluorescence.

Previous studies have suggested that lipids can change their behaviour within a cell depending on their fatty acid chain length [25]. Our studies demonstrate discrete neuroanatomical locations for gangliosides based on their carbon chain length. For example, within the hippocampus, the d18∶1 moiety of GM1, GD1 and GT1b appears to be expressed within the oriens layer, stratum radiatum and molecular layers; whereas the d20∶1 moiety is mainly restricted to the dentate gyrus. The findings are consistent with previous studies [13], [14] and provide a potential intriguing role for d20∶1 gangliosides in mediating functions of the subgranular zone within the dentate gyrus; a region where adult hippocampal neurogenesis occurs.

In this study, the change of d18∶1 and d20∶1 moieties were unique, especially within the cortex II ROI, a region that was based on Cresyl violet staining and depicts cortical tissue that was typically near the border of the infarct. In the case of GM1, GD1 and GT1b, although there was no tissue viability as assessed by the Cresyl violet staining, there was a strong signal of d20∶1 within the Cortex II ROI. In fact, the quantification data clearly demonstrated a strong induction of d20∶1 relative to d18∶1 for these gangliosides within the cortex II ROI (Figure 7). This finding suggests that different metabolic events are still occurring within the ‘infarcted tissue’. Moreover, given that the location of GM1 d20∶1 expression was located at the border of the infarcted tissue; it may suggest a potential role for the d20∶1 moiety in either infarct expansion or ‘sealing’ the region from further expansion. Conversely, the opposite trend was observed within the cortex II ROI for GM2 and GM3, whereby the d18∶1 moiety was induced at a greater level compared to d20∶1.

Combining the sensitivity of IMS with the superior anatomical resolution of immunofluorescence proved to be a valuable approach to examining the temporal profile of ganglioside expression following MCAO and long-term recovery. Our data demonstrate the expression of GM1 within the white matter fiber tracts and GD1a within the gray matter and are consistent with previous studies describing neuronal expression profiles of gangliosides [20], [21], [26]–[28]. The novel finding of GM2 by IMS and GM3 by both IMS and immunofluorescence may suggest the expression occurred within gray matter. This finding needs to be further explored with specificity, especially with respect to cell type expression patterns. One study has indicated a role for GM3 signaling within astrocytes [27], and thus it is possible that given GM3 expression timing following MCAO (3 and 7 d post-MCAO), GM3 may play an important role in neuroinflammatory mechanisms following stroke. In summary, the data suggest that brain region specific regulation of gangliosides, particularly with respect to sphingosine base length, may play a role in brain injury response to cerebral ischemia.

## Materials and Methods

### Animals

All procedures using animals were approved by the Institute for Biological Sciences Animal Care Committee (protocol 2007.13) following the guidelines established by the Canadian Council on Animal Care. C57BL/6 mice (20–23 g) were obtained from Charles River (St Foie, PQ, Canada).

### Unilateral Middle Cerebral Artery Occlusion (MCAO) and Reperfusion in mouse

Under temporary isofluorane anesthesia, mice were subjected to 30 min MCAO using an intraluminal filament as previously described [29], [30]. After 30 min of MCAO, the filament was withdrawn and blood flow was restored to basal levels, as assessed by laser Doppler flowmetry. Wounds were sutured following surgery. The body temperature of experimental mice was monitored before and after the MCAO surgery using a rectal probe and was maintained at 37°C using a heating pad and lamp. In preliminary experiments to verify a consistent stroke procedure, measurements of blood pressure, blood gases, and pH were also performed. Sham-operated mice were subjected to the same surgery without MCAO and used as controls. Mice were allowed to recover for 24 h, 3 d, 7 d, 14 d and 28 d following MCAO.

### Brain Tissue Processing

At various reperfusion time points, mice were anesthetized with isofluorane and sacrificed via decapitation. Fresh brains were isolated, flash frozen in dry ice and subsequently stored at −80°C. Ten µm coronal sections were cut using a Leica cryostat (Wetzlar, Germany) and sections were mounted on 41 mm×41 mm stainless steel MALDI slides (AB Sciex, Foster City, USA). Mounted sections were defrosted and dried *in-vacu* for an hour to prevent condensation. The plate was then placed in a humidor at ambient temperature at 80% relative humidity for 1 h. Sections were sprayed manually with 20 mg of matrix 5-chloro-2-mercaptobenzothiazole (CMBT) dissolved in 1 mL solution of chloroform, ethanol and water (4∶4∶1 v/v) using an airbrush. MALDI imaging was done on an Applied Biosystems 4800 MALID TOF/TOF. All analysis was done in negative ion using linear mode to obtain high detection sensitivity. The ionization was initiated with a third harmonic Nd:YAG pulse and 75–150 spectra were then summed to provide 1 pixel on the image. The pitch of the pixels was between 50–75 µm. The instrument was controlled via 4000 Series imaging software (Novartis, Mississauga, ON, Canada). Data analysis and visualization were done using TissueView (Novartis, Mississauga, ON, Canada).

### Quantitation of IMS

Regions of Interest (ROIs) were determined based on Cresyl violet staining. Mass spectra of several brain areas were measured, which included the infarcted cerebral cortex (cortex I), the peri-infarct cortex (the area adjacent to the infarcted cortex, labeled as Cortex II), the hippocampus and striatum. ROIs were also assessed for corresponding neuroanatomical regions within the contralateral hemisphere. Peaks for each species were measured independently. Total ion chromatographs were generated for each species and the signal noise was removed by subtracting the minimum intensity value from each peak. Each species peak was integrated to calculate the area underneath the peak curve. For calculation of the peak area, the most intense peak was chosen for each species, namely [GM1-H]^−1^, [GM2-H]^−1^, [GM3-H]^−1^, [GD1+K-2H]^−1^ and [GT1b+K-2H]^−1^. The mean peak areas were calculated for each surgical time point (n = 3) and expressed as the fold change from ipsilateral to contralateral ROIs. In the case where the contralateral ROI signal was higher than the ipsilateral ROI signal, the data was expressed as a fold decrease.

### Indirect Immunofluorescence Staining and Confocal Microscopy

At various reperfusion time points, mice were anesthetized with isofluorane and perfused transcardially, first with saline followed by 4% formaldehyde (pH 7.4). Brains were removed, post fixed in 4% formaldehyde (pH 7.4) for 18 h and cryoprotected in 30% sucrose for 36 h at 4°C. Coronal sections (10 µm thickness) were cut using a cryostat (Leica Microsystems, Wetzlar, Germany) and mounted on Superfrost slides (Fisher Scientific, Toronto, ON, Canada). Sections were rinsed 3 times in 10 mM PBS for 5 min each. Tissue sections were incubated with the following primary antibodies: mouse anti-GM1, mouse anti-GD1a (gifts kindly provided by Dr. Ronald Schnaar from the Johns Hopkins School of Medicine, Baltimore, USA) or mouse anti-GM3 (AMS Biotechnology, Lake Forest, CA, USA). Sections were incubated in primary antibody at a concentration of 1∶50 diluted in antibody buffer containing 10 mM PBS and 1.5% BSA in a humidified chamber overnight at 4°C. Sections were then washed 3 times with 10 mM PBS for 10 min. Sections were then incubated with a Rhodamine Red conjugated goat anti-mouse secondary antibody (Invitrogen, Carlsbad, CA, USA) at a concentration of 1∶600 diluted in antibody buffer for 1 h at room temperature. Sections were then washed 3 times in 10 mM PBS for 10 min, mounted with Dako fluorescent mounting media spiked with 2 µg/ml Hoechst 33258 (Sigma, Toronto, ON, Canada) to counter stain nuclei. Some sections omitted primary antibody incubation as a negative control. Confocal imaging was carried out on Olympus Fluoview FV1000 confocal laser scanning microscope (Olympus, Markham, ON, Canada). Imaging was performed with a 10× objective.

### Histology

Sections adjacent to those being imaged with MALDI were stained with Cresyl violet using a protocol as previously described [31] to observe regions of brain tissue viability loss.

### Statistical Analysis

Mean peak areas from individual ganglioside species were calculated and depicted as fold increase/decrease from ipsilateral to contralateral ROIs. ANOVA with a Dunnett's *posthoc* test was performed on each ROI comparison and statistical significance was achieved when p<0.05.
